# Salvaging the forgotten valve: T-TEER as the new frontier in torrential primary TR

**DOI:** 10.3389/fcvm.2026.1939762

**Published:** 2026-10-21

**Authors:** Nazario Carrabba, Mattia Alexis Amico, Andrea Taddei, Renato Valenti, Alfredo Giuseppe Cerillo, Pierluigi Stefano

**Affiliations:** 1 Department of Cardiothoracovascular Medicine, Careggi University Hospital, Firenze, Italy; 2 Cardiac Surgery Unit, Careggi University Hospital, Firenze, Italy; 3 Department of Geriatric and Intensive Care Medicine, Careggi University Hospital, Firenze, Italy; 4 Department of Clinical Experimental Medicine, University of Florence, Florence, Italy

**Keywords:** interventional echocardiography, right ventricular rupture, transcatheter tricuspid repair, tricuspid regurgitation, tricuspid valve

## Abstract

Tricuspid regurgitation (TR) has historically been considered the “forgotten valve disease”, but growing evidence has established its independent prognostic impact and has driven the rapid expansion of transcatheter tricuspid valve interventions. While transcatheter tricuspid edge-to-edge repair (T-TEER) has demonstrated safety and clinical benefit in patients with secondary functional TR, evidence supporting its use in primary TR remains scarce, and surgery continues to represent the standard treatment. However, many patients with primary TR are considered ineligible for surgery because of prohibitive operative risk, creating an important unmet therapeutic need. In this Perspective, we discuss the potential role of T-TEER in selected patients with primary TR through the paradigm of a patient who developed torrential TR following iatrogenic rupture of the septal papillary muscle–chordal apparatus during cardiac device lead extraction. Owing to multiple comorbidities and prohibitive surgical risk, the patient underwent successful transcatheter repair with two PASCAL Ace devices, achieving durable TR reduction and sustained clinical improvement at one-year follow-up. Building on this experience, we review the current evidence supporting transcatheter tricuspid interventions, discuss the anatomical and procedural considerations that may enable T-TEER in selected forms of primary TR, and highlight the limitations of the available literature. We propose that carefully selected anatomical subsets of primary TR may represent an emerging frontier for transcatheter therapies, bridging the gap between guideline-directed management and individualized treatment when surgery is not feasible. Prospective studies are warranted to define patient selection criteria, procedural feasibility, and long-term clinical outcomes.

## Introduction

For many years, tricuspid regurgitation (TR) was considered the “forgotten valve disease”, receiving limited clinical attention compared with left-sided valvular disorders. This historical underestimation was largely driven by the perception of TR as a benign consequence of other cardiac conditions rather than an independent pathological entity. However, accumulating evidence has demonstrated that significant TR is associated with progressive right ventricular remodeling, systemic congestion, impaired quality of life, and increased mortality. These findings have led to a substantial reappraisal of the disease and to a growing recognition of the prognostic impact of TR in contemporary clinical practice.

Recent guideline updates have reflected this paradigm shift by expanding the indications for intervention and emphasizing the importance of timely treatment before the development of irreversible right ventricular dysfunction and end-organ damage. The rapid development of transcatheter tricuspid valve interventions, which have expanded therapeutic opportunities for patients previously considered unsuitable for surgery, have demonstrated encouraging results in reducing TR severity and improving symptoms and quality of life, establishing a new therapeutic era for severe tricuspid valve disease. Both tricuspid transcatheter edge-to-edge repair (T-TEER) and tricuspid transcatheter valve replacement (TTVR) have transformed the therapeutic landscape.

According to current ESC 2025 guidelines (1) transcatheter treatment is mainly indicated for patients with secondary functional TR with high or prohibitive surgical risk, which represents the predominant phenotype investigated in contemporary clinical trials. However, recently encouraging data were reported by dedicated study evaluating T-TEER in primary TR, including prolapse and flail leaflet phenotypes (2, 3). Surgical repair or replacement remains the standard therapeutic approach for primary TR; however, for patients not suitable for surgery due to advanced comorbidities, previous cardiac surgery, or excessive operative risk, percutaneous approach may be an alternative therapeutic option.

Among the different causes of primary TR, iatrogenic damage of the tricuspid valve apparatus represents a particularly challenging and poorly explored entity. Traumatic primary TR with disruption of papillary muscles or leaflet structures may result in torrential TR with progressive right ventricular remodeling and dysfunction, while therapeutic strategies remain largely based on individualized clinical decisions rather than robust evidence.

In this Perspective article, we discuss the potential role of T-TEER in selected patients with primary TR through the paradigm of an iatrogenic septal papillary muscle rupture successfully treated with transcatheter repair. We propose that, although current ESC guidelines are predominantly focused on secondary TR, new evidences are emerging for primary TR and carefully selected anatomical subsets of primary traumatic TR may represent an emerging frontier for transcatheter therapies in this challenging population.

## Salvaging the forgotten valve: T-TEER as the new frontier in torrential primary TR

In 2011, a 52-year-old woman was evaluated following a witnessed out-of-hospital sudden cardiac arrest. Her medical history included tobacco use and severe obesity. Emergency medical services performed cardiopulmonary resuscitation and delivered appropriate DC shocks, achieving return of spontaneous circulation. Upon admission to the emergency department, echocardiography revealed a severely dilated left ventricle with markedly impaired systolic function and mild mitral regurgitation. Emergent coronary angiography excluded obstructive coronary artery disease. Based on these findings, a diagnosis of new-onset non-ischemic dilated cardiomyopathy was established. The patient was commenced on optimal guideline-directed medical therapy available at the time. Given the occurrence of sudden cardiac arrest, the severely reduced left ventricular ejection fraction (LVEF), and the presence of complete left bundle branch block on electrocardiogram, a cardiac resynchronization therapy defibrillator (CRT-D) was implanted for secondary prevention and heart failure management. Genetic testing for common pathogenic variants associated with dilated cardiomyopathy was negative.

In the months following patient discharge, serial echocardiographic assessments documented a progressive and near-complete recovery of LVEF. However, her clinical course over the next year was complicated by multiple inappropriate shocks delivered by CRT-D during periods of physical activity, triggered by episodes of paroxysmal supraventricular tachycardia and atrial fibrillation, requiring several device reprogramming to resolve.

In 2018, at the age of 58, she was diagnosed with early-stage left breast cancer and was treated with surgery followed by adjuvant chemotherapy. Due to the need for left-sided chest irradiation, CRT-D system explantation and re-implantation on the right side was planned. During the percutaneous lead-extraction procedure, the patient developed cardiac tamponade due to right ventricular (RV) free wall perforation. She underwent successful emergent surgical repair with patch closure. During the same hospitalization, postoperative echocardiography revealed a flail segment of the septal tricuspid leaflet causing torrential TR. Transesophageal echocardiography confirmed torrential TR due to rupture of the septal papillary muscle–chordal apparatus, resulting in loss of leaflet support and marked leaflet flail. The findings were consistent with an iatrogenic injury of the tricuspid sub-valvular apparatus occurring during the lead extraction procedure.

Given the recent emergent cardiac surgery, the oncologic diagnosis, and the need to prioritize cancer treatment, surgical intervention for TR was deferred in favor of a conservative medical approach with high-dose diuretics, achieving temporary clinical stabilization and control of systemic congestion related to torrential TR. The postoperative course was also complicated by Dressler's syndrome. She subsequently underwent successful CRT-D reimplantation on the right side and completed the planned radiotherapy for breast cancer. Follow-up oncologic evaluations confirmed stable disease with no recurrence.

During cardiology follow-up from 2019 to 2023, the patient remained symptomatic with persistent massive TR and apparently preserved RV function, requiring high-dose diuretics. TR severity was evaluated using *vena contracta* width, size of the regurgitation area, hepatic vein flow patterns as well as inferior vena cava variability (see Table 1). Moreover, during these years, chronic back pain led to reduced mobility and weight gain, further compromising her clinical condition. The presence of a patch on the RV free wall may potentially mimic a restrictive physiology, impairing distensibility and altering the ventricular response to volume loading. In this condition, it is well known that the pressure-volume relationship is markedly non-linear; specifically, minimal increments in end-diastolic volume lead to intraventricular pressure rises. This lack of distensibility forces volume upstream, causing right atrial and caval overload, congestive hepatopathy, and visceral edema, mechanisms typically refractory to medical therapy. The patient exhibited all these features: echocardiography showed a dilated inferior vena cava with poor inspiratory collapse and systolic flow reversal in the hepatic veins, accompanied by peripheral oedema and persistently elevated transaminase levels, supporting this hypothesis. Furthermore, the combination of a rigid RV and torrential TR collapses right-sided forward flow, inducing an LV “starvation” state. Despite preserved contractility, the LV receives insufficient preload to maintain stroke volume via the Frank-Starling mechanism. This serial ventricular interdependence may generate a potential low-output state explaining the persistent hypotension and fatigue observed despite optimal management.

**Table 1:** Integrated pre-operative echocardiographic assessment of cardiac chambers and tricuspid regurgitation severity.

Right sided-chambers	Patient value	Reference value
RV basal diameter	37 mm	< 42 mm
RV mid-diameter	27 mm	< 36 mm
RV EDVi	27 mL/m^2^	≤ 95 mL/m^2^
RV ESVi	11 mL/m^2^	≤37 mL/m^2^
TAPSE	14 mm	≥17 mm
RV TDI S’	8 cm/s	≥10 cm/s
RV FAC	31%	> 35%
3D RVEF	38%	≥ 50%
RV free-wall longitudinal strain	- 18%	≥ −23%
RA area	23 cm^2^	Increased RA size

Tricuspid regurgitation	Patient value	Criteria for severe TR
EROA	63 mm^2^	≥40 mm^2^
Biplane vena contracta width	23 mm	≥7 mm; >21 mm = torrential

Systemic venous	Patient value	Reference value
Hepatic vein flow	Systolic flow reversal	No Systolic flow reversal
Biplane vena contracta width	25 mm and 35% collapse	≤21 mm and >50% collapse

At the 2024 follow-up evaluation, the patient presented with escalating exertional dyspnea and systemic venous congestion, indicative of progressive right-sided heart failure. Transthoracic echocardiography documented worsening torrential TR due to the previously established iatrogenic rupture of the septal papillary muscle–chordal apparatus. Despite optimized medical therapy, symptoms related to right-sided volume overload progressively worsened. Following a multidisciplinary Heart Team consensus, conventional tricuspid valve surgery was deemed prohibitive due to the cumulative perioperative burden (STS-PROM: 16%). Moreover, the orthotopic valve was not available at our Institute at that time. Considering the favorable anatomical features of the septal leaflet flail, with sufficient leaflet tissue for grasping and a permissive coaptation gap (<8.5 mm) as demonstrated by transgastric and deep mid-esophageal TEE views, T-TEER was therefore pursued as a tailored, minimally invasive therapeutic alternative, facilitated by favorable lead positioning. Heart Team decision-making pathway is resumed in Figure 1.

**Figure 1:**
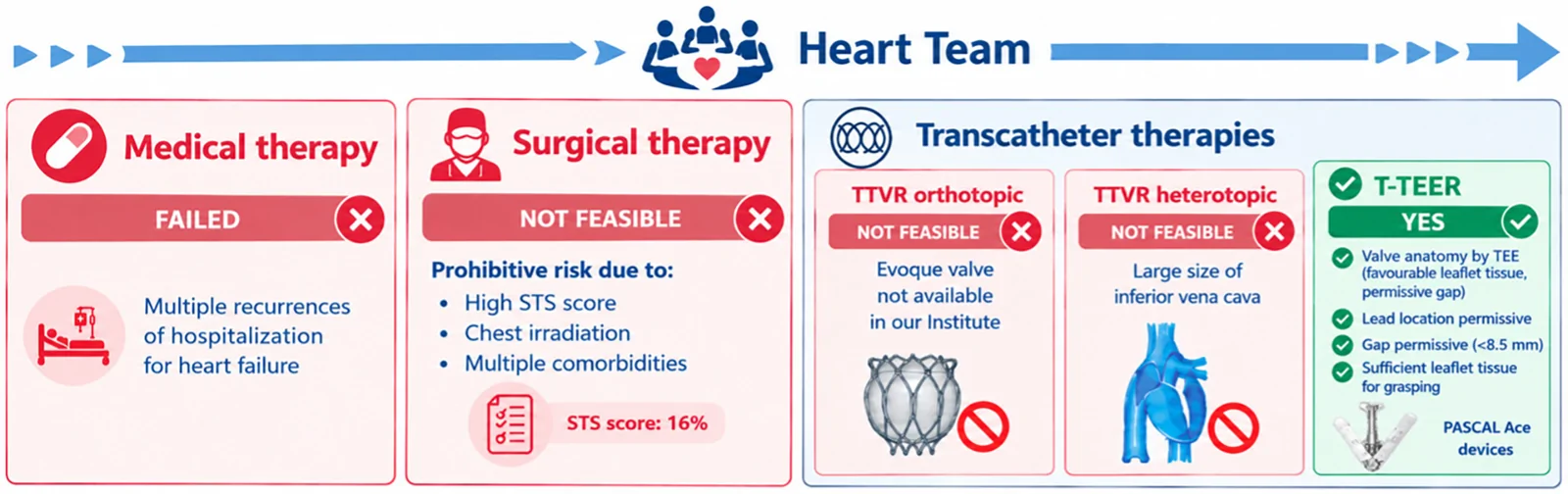
Heart team shared decision-making pathway.

In January 2025, the patient underwent T-TEER to correct torrential TR caused by septal leaflet flail due to iatrogenic rupture of the septal papillary muscle–chordal apparatus. Intraprocedural echo guidance was performed using biplane trans-gastric and deep mid-esophageal 2-dimensional transesophageal views. The procedure was performed using two *PASCAL Ace devices* (Figure 2 and Supplementary Data). Compared with the original PASCAL system, the PASCAL Ace device features a elongation capability, a central spacer, and independent leaflet clasping, facilitating device positioning and leaflet capture in challenging tricuspid valve anatomies. Both devices were placed between the septal and anterior leaflets. The first device was implanted anterior to the target lesion to stabilize the valve anatomy and to avoid shadow effect, allowing for the subsequent positioning of the second clip at the site of the septal leaflet flail. Both devices were placed using independent leaflet capture. As expected, after the first device placement the correction of the TR was not sufficient. After the second device placement, an effective correction of the underlying mechanism responsible for torrential TR was obtained, with an excellent intraprocedural outcome and only trivial residual TR and a mean gradient of 5 mmHg. The procedure was uncomplicated, and after five days the patient was discharged. Pre-discharge echocardiography showed only mild residual TR between the septal and anterior leaflets.

**Figure 2:**
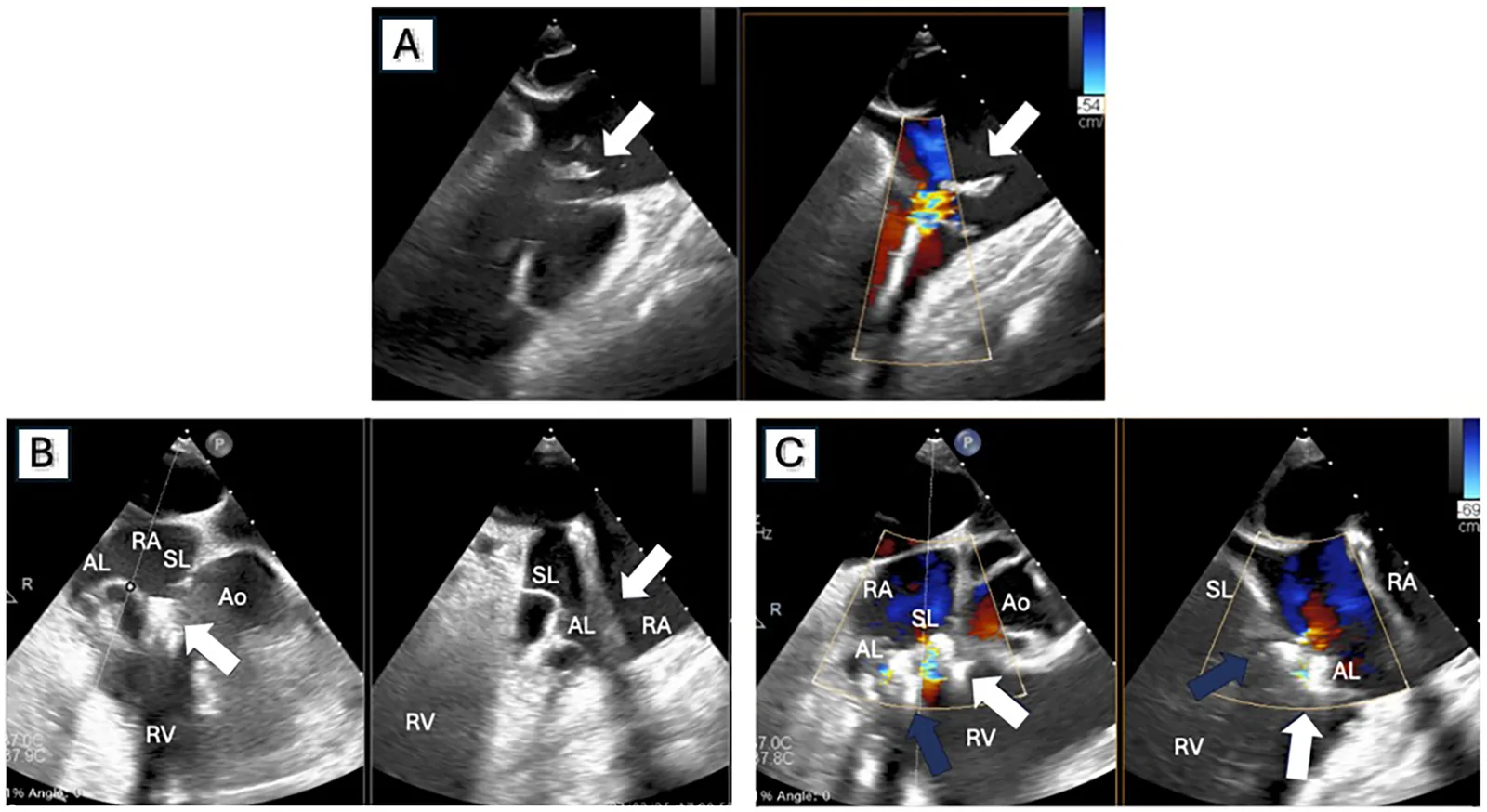
Intraoperative transesophageal echocardiography. **(A)** Mid-esophageal right ventricular inflow view without and with color-Doppler, showing septal leaflet flail with an attached portion of papillary muscle (white arrow) causing massive tricuspid regurgitation. **(B)** Mid-esophageal right ventricle inflow/outflow view with orthogonal multiplane, showing the first PASCAL Ace device implanted between the septal and the anterior leaflet (white arrow). **(C)** Mid-esophageal right ventricle inflow/outflow view with orthogonal multiplane, showing color Doppler imaging following the placement of the second PASCAL Ace device between the septal and the anterior leaflet in a more central position (blue arrow), demonstrating a mild residual tricuspid regurgitation. AL, anterior leaflet; Ao, aorta; SL, septal leaflet; RA, right atrium; RV, right ventricle.

At 1-year follow-up, the patient was well compensated, and transthoracic echocardiography confirmed a durable result, with only mild residual tricuspid regurgitation and a mean transvalvular gradient of 5 mmHg, without evidence of clinically significant tricuspid stenosis. There was no evidence of leaflet flail, and the devices remained well positioned with no signs of leaflet detachment. Regarding functional RV parameters, no significant changes were observed in tricuspid annular plane systolic excursion, fractional area change, or RV strain. A significant reduction in right atrial volume was observed, whereas RV end-diastolic diameter remained unchanged.

The patient's clinical timeline is summarized in Figure 3.

**Figure 3:**
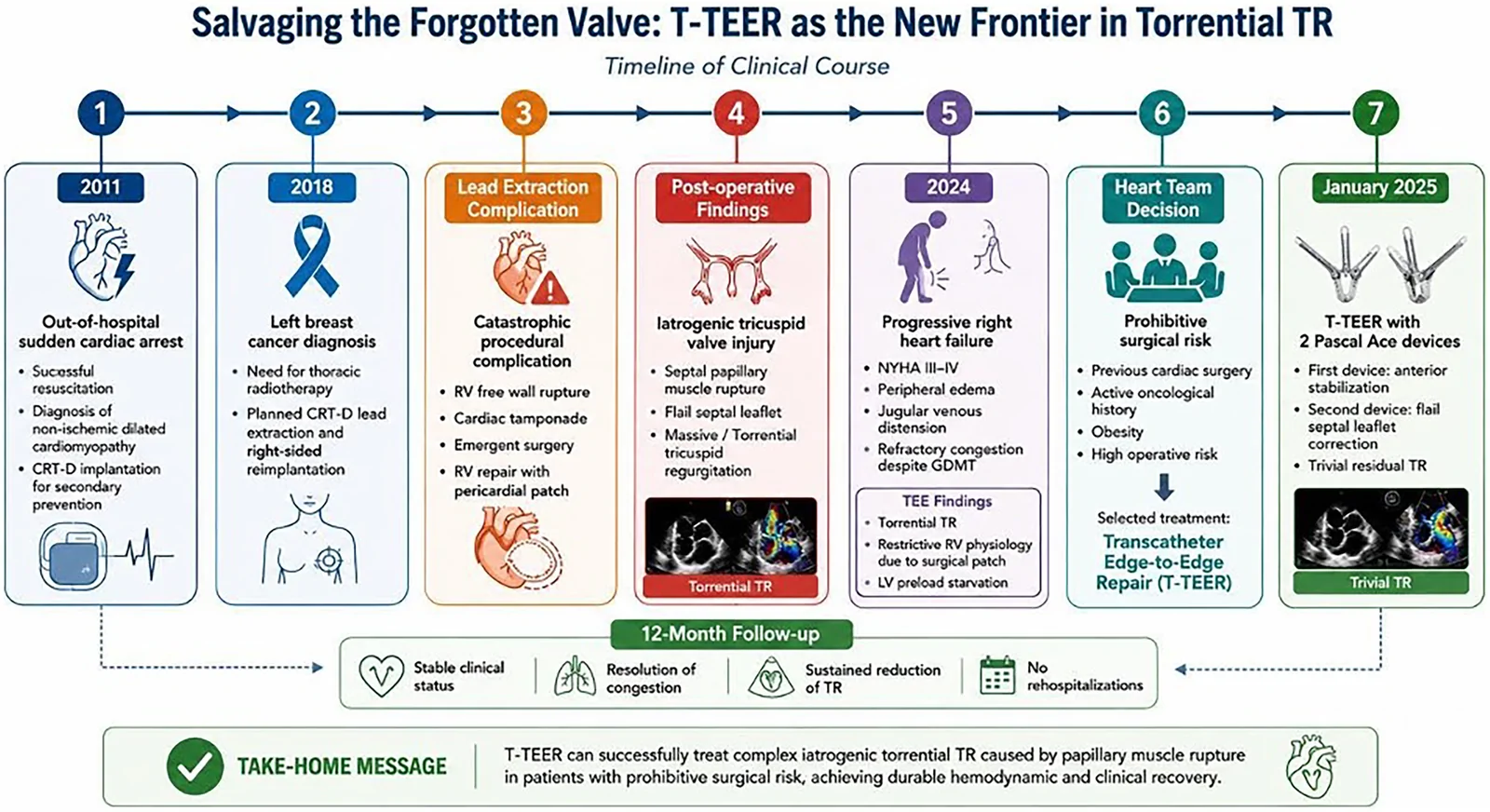
Patient timeline.

## Discussion

### Etiology and pathophysiology of primary isolated TR

Tricuspid regurgitation can be classified as primary, resulting from intrinsic abnormalities of the tricuspid valve apparatus, or secondary, resulting from right ventricular or annular remodeling. Primary TR can be caused by endocarditis, degenerative prolapse, cardiac device lead-related injury, prosthetic valve dysfunction, rheumatic heart disease, carcinoid heart disease, or congenital abnormalities such as Ebstein's anomaly (4). Infective endocarditis, particularly in intravenous drug users or patients with indwelling catheters, can lead to leaflet destruction or perforation (5). Prosthetic valve regurgitation, whether due to degeneration, thrombosis, or paravalvular leaks, can also cause significant TR, often requiring reintervention (6).

Cardiac implantable electronic devices (CIEDs), including implantable cardioverter defibrillators, permanent pacemakers, and cardiac resynchronization therapy devices, represent an increasingly recognized cause of isolated TR. Variable degrees of lead-induced TR ranging from 7% to 45% have been reported in patients with CIEDs (7). The mechanisms underlying CIED-induced TR can be categorized into implantation-related, pacing-induced, and device-mediated factors (7). However, most CIED-related TR cases are caused by leaflet interference, impingement, or entanglement, whereas direct traumatic disruption of the tricuspid sub-valvular apparatus represents an exceptionally rare mechanism.

Iatrogenic rupture of the tricuspid sub-valvular apparatus during cardiac device lead extraction represents an extremely rare mechanism of primary TR. In our patient, lead extraction was complicated by right ventricular free wall perforation requiring emergent surgical repair, with associated injury of the tricuspid papillary muscle–chordal apparatus resulting in torrential TR. The pathophysiology of primary TR involves direct damage or malformation of the valve apparatus, leading to progressive right ventricular volume overload, elevated right atrial pressure, systemic venous congestion, and eventually right-sided heart failure (8). Over time, chronic volume overload may result in right ventricular dilatation, impaired RV function, and worsening clinical status (6). Interestingly, in our patient, surgical repair of the RV free wall injury may have contributed to maintaining RV geometry despite the chronic volume overload imposed by severe TR.

### Management of isolated TR

Surgical intervention remains the reference treatment for severe primary TR in selected patients, particularly in the presence of symptoms, progressive right ventricular remodeling, or concomitant left-sided valve surgery. Despite the increasing prevalence of TR and its association with poor survival, isolated tricuspid valve surgery remains infrequently performed (9). To address this unmet clinical need, several less invasive transcatheter tricuspid valve therapies have emerged as alternative treatment options for high-risk patients with severe functional TR (9).

T-TEER devices aim to improve leaflet coaptation by directly approximating the leaflets or indirectly by addressing annular dilatation. TTVR can be orthotopic, consisting of prosthetic valve implantation in the anatomical position of the tricuspid annulus, or heterotopic, involving implantation in a different anatomical location to mitigate the hemodynamic consequences of severe TR.

In a study involving 2,413 patients with severe isolated functional TR, participants were divided into three groups: 1,217 were conservatively managed, 551 underwent isolated TV surgery, and 645 received T-TEER. Survival over two years served as the primary endpoint. The TRI-SCORE, which assesses the severity of TR, showed 32% of patients with a low TRI-SCORE (≤3), 33% had an intermediate score (4–5), and 35% had a high score (≥6). Successful correction rates were high, with 97% in the surgical group and 65% in the transcatheter group. Survival rates decreased across all treatment modalities with increasing TRI-SCORE severity (10). The study concluded that, while survival decreased with higher TRI-SCOREs across all treatment modalities, early and successful surgical or transcatheter interventions significantly improved two-year survival rates in patients with low and, to a lesser extent, intermediate TRI-SCOREs, but no improvement was seen in those with high scores (10).

Orban et al. (11) reported a dual-center observational study of 50 patients with severe TR and right heart failure, including 14 with isolated TR and 36 with combined MR and TR. At 6 months, 90% had a reduction in TR severity and 79% improved their NYHA class, with significant improvements in 6-minute walk distance (+84 m), NTproBNP (−30%), and quality of life. Outcomes were similar between isolated and combined procedures, although eight deaths and 14 heart failure hospitalizations occurred during follow-up.

In a subsequent study of 119 patients undergoing isolated transcatheter TV repair, Orban et al. (12). Reported successful reduction of TR to moderate or less, with 72% maintaining this result at 1 year. The procedure was associated with a 22% reduction in annual heart failure hospitalization and significant improvements in NYHA class, 6-minute walk distance, and quality of life. Procedural success was also associated with improved 1-year survival and event-free survival.

The CLASP TR Early Feasibility Study, a single-arm, multicenter, prospective study, assessed the 1-year outcomes of the Edwards PASCAL transcatheter valve repair system in patients with severe or greater TR who remained symptomatic despite medical treatment. The study involved 65 patients with a mean age of 77.4 years (13). Initial results showed a cardiovascular mortality of 3.1%, a stroke rate of 1.5%, and no device-related reinterventions within the first 30 days. From 30 days to one year, there were additional outcomes, including three cardiovascular deaths (4.8%), two strokes (3.2%), and one unplanned reintervention (1.6%). By the end of the first year, TR severity had significantly decreased, with 86.0% of patients achieving moderate or less TR and all patients experiencing at least one grade reduction in TR severity. Kaplan–Meier analysis showed freedom from all-cause mortality and heart failure hospitalization at 87.9% and 78.5%, respectively. There was a notable improvement in NYHA functional class (92% in class I or II), a 94 m increase in 6-minute walk distance, and an 18-point improvement in Kansas City Cardiomyopathy Questionnaire scores (13).

A meta-analysis performed by Bocchino et al., including fourteen studies and more than 770 patients who underwent isolated transcatheter TV repair, reported significant improvements in NYHA functional status, 6-minute walking distance, and TR severity reduction. Patients had a mean age of 77 years, a mean European System for Cardiac Operative Risk Evaluation score of 6.8%, and a mean follow-up of 7 months (14).

The TRILUMINATE pivotal trial showed that tricuspid TEER compared with medical therapy alone was safe in patients with severe TR, reduced TR severity, and was associated with improvement in quality of life (15). Similarly, results from TRISCEND II showed that among patients with severe symptomatic TR, orthotopic transcatheter tricuspid valve replacement using the EVOQUE system combined with medical therapy was superior to medical therapy alone in improving cardiovascular outcomes, primarily through improvements in symptoms and quality of life, without significant differences in mortality or heart failure hospitalization (16). These findings suggest that current transcatheter therapies provide primarily symptomatic and functional benefits rather than proven survival advantages. TR reduction appears greater with TTVR, and future head-to-head comparisons will be important.

In February 2024, the U.S. Food and Drug Administration (FDA) approved the Edwards EVOQUE Tricuspid Valve Replacement System, the first FDA-approved transcatheter tricuspid valve replacement system. The EVOQUE system features a self-expanding nitinol frame with bovine pericardial leaflets and an intra-annular sealing skirt, delivered transfemorally via a 28F delivery system (17). In April 2024, the FDA approved Abbott's TriClip Transcatheter Edge-to-Edge Repair System, offering a minimally invasive option for selected patients with symptomatic severe TR at high surgical risk. The TriClip G4 System utilizes TEER technology, approximating portions of the tricuspid valve leaflets to reduce regurgitation without the need for open-heart surgery. Both devices represent significant advancements in the minimally invasive treatment of severe TR, providing new therapeutic options for patients who are at high surgical risk or considered unsuitable for conventional surgery (15, 16).

Dannenberg et al. (2), evaluated 201 patients, of whom 27 (13.4%) were classified as primary TR and 174 (86.6%) as TR of secondary origin. Primary TR was defined as tricuspid valve (TV) prolapse or flail TV leaflets as assessed by transesophageal echocardiography. The authors concluded that T-TEER achieves a robust TR reduction in primary TR patients 1 year after intervention with noninferior clinical results to treatment for secondary TR regarding mortality, re-hospitalization, and NYHA functional class.

Sugiura et al. (3) evaluated 114 patients with primary TR undergoing T-TEER, demonstrating the effective reduction in primary TR severity, leading to right heart reverse remodeling and meaningful symptomatic relief. Both studies provide encouraging results and may expand the therapeutic role of minimally invasive transcatheter interventions beyond secondary/functional TR to selected primary degenerative etiologies.

The recent European Society of Cardiology 2025 guidelines for the management of valvular heart disease (1) emphasized the prognostic relevance of TR and the importance of intervention before irreversible right ventricular damage develops. Surgical treatment remains the cornerstone for severe primary and secondary TR in appropriate candidates, while transcatheter approaches may be considered in selected high-risk patients with suitable anatomy and absence of advanced pulmonary hypertension or severe RV dysfunction.

### Beyond secondary TR: expanding the role of T-TEER in selected primary TR

Current ESC guidelines recommend transcatheter tricuspid interventions for patients with secondary functional TR with high or prohibitive surgical risk. Recently, encouraging data has emerged supporting T-TEER in selected patients with primary TR. Primary TR encompasses heterogeneous anatomical conditions, including localized structural lesions of the leaflet or sub-valvular apparatus that may be technically suitable for transcatheter repair.

Our case represents an example of a highly selected form of primary TR caused by iatrogenic rupture of the tricuspid papillary muscle–chordal apparatus, in which conventional surgery was considered prohibitive due to the cumulative clinical burden. The successful use of T-TEER suggests that, beyond secondary functional TR, selected primary TR anatomies may represent an emerging field for transcatheter therapies.

Future studies should focus on defining anatomical criteria for procedural feasibility, identifying predictors of durable repair, and establishing long-term clinical outcomes in patients with primary TR treated with transcatheter approaches. This evolving perspective may contribute to expanding the therapeutic paradigm of tricuspid valve disease beyond the traditional distinction between surgical treatment and conservative management.

## Conclusion

After multidisciplinary Heart Team evaluation, T-TEER may represent a tailored therapeutic option in patients with torrential primary TR with prohibitive surgical risk, bridging the gap between guideline-directed recommendations and individualized clinical decision-making. Selected anatomical subsets of primary TR may represent an emerging field for transcatheter intervention, although further studies are required to define patient selection criteria and long-term outcomes.

## Data Availability

The raw data supporting the conclusions of this article will be made available by the authors, without undue reservation.

## Supplementary material

Supplementary Video 1 Mid-esophageal right ventricular inflow view without and with color-Doppler, showing septal leaflet flail with attached a portion of papillary muscle (white arrow) causing massive tricuspid regurgitation.

Supplementary Video 2 Mid-esophageal right ventricle inflow/outflow view with orthogonal multiplane, showing the first Pascal-Ace device implanted between the septal and the anterior leaflet (white arrow).

Supplementary Video 3 Mid-esophageal right ventricle inflow/outflow view with orthogonal multiplane, showing color Doppler imaging following the placement of the second Pascal Ace device between the septal and the anterior leaflet in a more central position (blue arrow), demonstrating a mild residual tricuspid regurgitation.
